# Study on Microstructure Evolution Mechanism of Gradient Structure Surface of AA7075 Aluminum Alloy by Ultrasonic Surface Rolling Treatment

**DOI:** 10.3390/ma16165616

**Published:** 2023-08-14

**Authors:** Lei Fu, Xiulan Li, Li Lin, Zhengguo Wang, Yingqian Zhang, Yunrong Luo, Shisen Yan, Chao He, Qingyuan Wang

**Affiliations:** 1 School of Mechanical Engineering, Sichuan University of Science & Engineering, Zigong 643000, China; kunmingfulei@126.com (L.F.); hhqlxl@163.com (X.L.); wang_guo860@163.com (Z.W.); luoyunrong2004@aliyun.com (Y.L.); shisenyan@163.com (S.Y.); 2 Failure Mechanics and Engineering Disaster Prevention, Key Lab of Sichuan Province, Sichuan University, Chengdu 610065, China; 3 Key Laboratory of Deep Earth Science and Engineering (Sichuan University), Ministry of Education, Chengdu 610065, China; 4 Vanadium and Titanium Resource Comprehensive Utilization Key Laboratory of Sichuan Province, Panzhihua 617000, China; linli1031@126.com; 5 Sichuan Provincial Key Lab of Process Equipment and Control, Zigong 643000, China; 6 School of Civil Engineering, Sichuan University of Science & Engineering, Zigong 643000, China; zyq13568328215@163.com

**Keywords:** AA7075 aluminum alloy, ultrasonic surface rolling treatment, microstructure evolution mechanism, gradient structure

## Abstract

The materials with grain size gradient variation on the surface, which were prepared with mechanical-induced severe plastic deformation, always show high resistance to high and low cycle fatigue and frictional wear because of their good strength–ductility synergy. The ultrasonic surface rolling treatment (USRT) has the advantages of high processing efficiency, good surface quality, and large residual compressive stress introduced to the surface after treatment. The USRT was used to prepare aluminum alloy (AA7075) samples with a surface gradient structure; meanwhile, the microstructural evolution mechanism of the deformation layers on the gradient structure was studied with XRD, SEM, and TEM. The microstructure with gradient distribution of grain size and dislocation density formed on the surface of AA7075 aluminum alloy after USRT. The surface layer consists of nanocrystals with random orientation distribution, and high-density dislocation cells and subgrains formed in some grains in the subsurface layer, while the center of the material is an undeformed coarse-grained matrix. The results show that the dislocation slip dominates the grain refinement process, following the continuous cutting and refinement of dislocation cells, subgrains, and fragmentation of the second precipitates. This study systematically clarified the mechanism of grain refinement and nanocrystallization on the surface of high-strength aluminum alloys and laid a theoretical foundation for further research on mechanical behavior and surface friction and wear properties of high-strength non-ferrous materials with gradient structure.

## 1. Introduction

**Figure 1:**
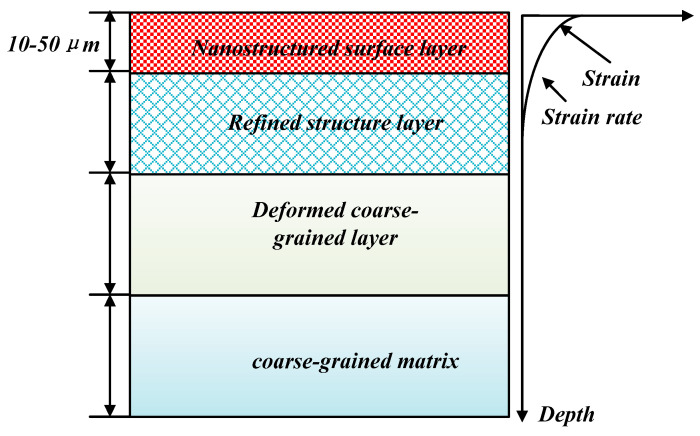
Schematic showing internal microstructure of gradient structure materials [4].

The failure of materials due to fatigue, frictional wear, and corrosion often originates from the material surface during their service life. Optimized surface properties of materials can prolong their service life [1,2,3]. Lu et al. [4] prepared materials by mechanically inducing severe plastic deformation on the material surface, resulting in a uniform gradient grain size from the surface to the core, as shown in Figure 1. In the last decade, many investigations have been reported where the gradients of composition and structure were targeted to improve the properties of materials [5,6,7]. Currently, the methods for preparing gradient structured materials include ultrasonic impact treatment (UIT), surface mechanical attrition (SMAT), surface mechanical grinding treatment (SMGT) [8], and surface mechanical rolling treatment (SMRT) [9].

These methods can be applied to prepare various gradient structure materials, such as gradient nanograins, nanolayers, and nanotwins [8,10,11,12,13,14,15,16]. The gradient structure of a metal surface consists of nanograins, while the core is an undeformed coarse-grained matrix.

The grain size, grain boundaries, and other interfacial properties show a gradient variation with depth, and the mechanical properties (such as strength and hardness) also exhibit a gradient distribution in space. The performance of the structure is superior to that of coarse-grained and uniform bulk nanostructured materials with the same composition [17,18,19]. In contrast to the simple mixing or compounding of nanocrystals, submicron crystals, microcrystals, and coarse crystals, the surface grain size of the gradient structure material exhibits a gradual gradient distribution. Previous research has shown that the deformation twinning plays a key role in refining grains to the nanometer scale via severe plastic deformation. Randomly orientated nanoscale grains result from the interaction between dislocations and numerous twin boundaries at the nanoscale, and dislocation slip dominates the grain refinement process [20,21,22]. This distinct form of a structure can reflect unique performance characteristics corresponding to specific characteristic sizes. For instance, the material exhibits good comprehensive properties during service [22,23,24], which provide good fatigue resistance and fracture performance as well as excellent resistance to strain hardening ability, resistance to abrasion, and corrosion resistance. Therefore, the applications of gradient structure materials are diverse, and they constitute one of the thriving fields of research in materials engineering.

Compared with the conventional surface treatment techniques, such as shot peening (SP), ultrasonic peening (USSP), and surface rolling (SR), ultrasonic surface rolling treatment (USRT) offers many advantages, such as high processing efficiency, good surface quality, and large residual compressive stress induced on the surface after treatment. Additionally, it can be used to treat high-strength materials. In this research, the newly developed ultrasonic surface rolling process (USRP) was used to prepare AA7075 samples with a surface gradient structure. The microstructure of the deformation layer of the gradient structure was characterized with scanning electron microscopy (SEM), X-ray diffraction (XRD), and transmission electron microscopy (TEM). Compared with the AA7075 samples treated with USSP [25,26], the gradient structure of the AA7075 samples prepared with USRT has a thicker surface deformation layer, a deeper surface hardening layer, and better surface quality. The results of this study lay the foundation for future studies to investigate mechanical properties, fatigue properties, surface friction, and wear properties. This research also provides a theoretical foundation for studying the surface grain refinement and nanocrystallization of the same type of precipitation-strengthened aluminum alloy.

## 2. Experimental

### 2.1. Materials and Sample Preparation

The specimens were machined from the commercial AA7075 aluminum alloy hot-forged plate (thickness: 20 mm, aluminum content: 99%, impurity content: 0.1%) produced by Southwest Aluminum (Group) Co., LTD., Chongqing, China. The ingot was heated to approximately 420 °C, forged through a 6000 tons press, oil-quenched at 470 °C, subjected to heat preservation for 120 min, water-quenched, and then heated to 120 °C for 4 h. Finally, the specimens were treated with heat preservation for 24 h. The chemical compositions and mechanical properties are listed in Table 1 and Table 2, respectively.

**Figure 2:**
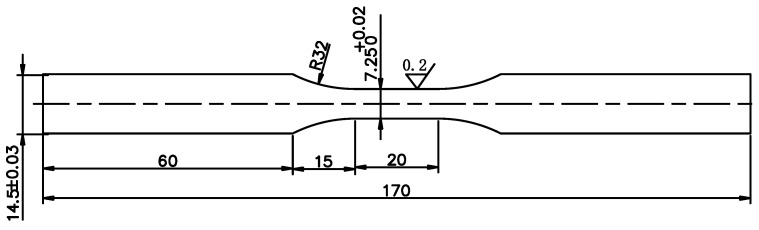
The geometric dimensions of the sample.

The hourglass-shaped specimens (with a gage length of 20 mm and a central diameter of 7.25 mm) were prepared in the long traverse (LT) direction, as shown in Figure 2. On the CNC machine tool, the outer circular surface of the gauge length part of the specimens was polished step-by-step with different grit water-abrasive paper, and then the surface scratch was removed with mechanical polishing with 3.5 μm diamond grinding paste until a mirror finish was achieved.

### 2.2. Analysis of the USRT

**Figure 3:**
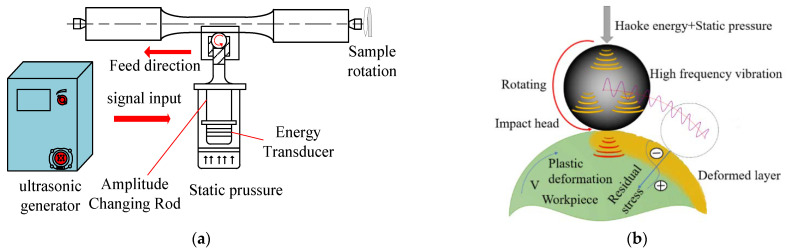
Diagram showing working principle of ultrasonic rolling treatment [27]: (**a**) Schematic showing working principle and (**b**) partial enlarged drawing of the contact position between the rolling head and the workpiece.

In this study, the USRT (HK30C Haoke, Shandong Huayun electromechanical Technology Co., LTD, Jinan, China) was carried out to fabricate a gradient nanostructured surface layer on the aluminum alloy sample. The main components of the USRT system include the actuator, controller, horns, impact heads, and ultrasonic generators. The working principle of USRT is explained in Figure 3 [27]. First, the electric energy of the ultrasonic generator is converted into ultrasonic vibration using a piezoelectric ceramic transducer. The ultrasonic vibration energy is then amplified by the horn and finally transformed into ultrasonic impact (tens of thousands of times per second) on the workpiece. At the same time, a cemented carbide rolling ball is designed at the front end of the tool head, and a certain static pressure is applied to the rolling ball. Eventually, the surface of the workpiece is treated with the combination of static load rolling and ultrasonic impact energy. USRT has high possessing efficiency, and the processed workpieces produce low surface roughness, a deep deformation layer, and a thick surface hardening layer.

The rolling parameters of AA7075 were decided by fine-tuning the processing parameters, such as the sample speed, the feeding speed of the rolling head, the indentation depth of the rolling head, and the number of rolling treatments. The USRT processing parameters are listed in Table 3.

### 2.3. Microstructural Characterization

#### 2.3.1. Sample Preparation

**Figure 4:**
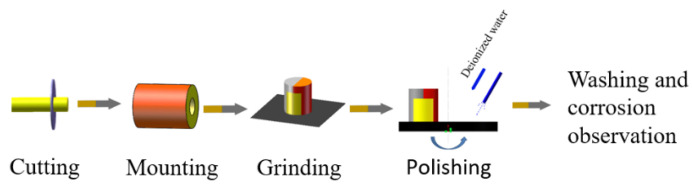
Schematic diagram of sample preparation process for TEM.

The samples for microstructure observation were prepared by cutting, producing a protective electroplating layer, grinding, and finally polishing the surfaces. (1) Sampling: First, a cylindrical sample (φ7.25 × 15 mm) was cut from the gage length of the original coarse crystal. The sample for USRT was prepared with wire electrical discharge machining (WEDM). (2) Protective platinum plating: First, the cut surfaces of the cylindrical metallographic sample were polished with 450# sandpaper, then cleaned with acetone, and then dried with a hair dryer. To prevent the effect of a chamfer on the microstructure, a deformed layer was produced during the subsequent grinding and polishing processes. The electrochemical plating method was used to generate a pure platinum protective layer with a thickness of approximately 200 μm. (3) Mounting: A mounting machine was used to cold mount the experimental samples. (4) Grinding and polishing: The sample was polished step-by-step with 250#, 400#, 800#, 1200#, and 2000# watermill sandpapers and then polished on the machine by applying a diamond grinding paste with a particle size of 3.5 μm using grinding discs. (5) Washing and drying: The sample was placed in an acetone solution, cleaned using an ultrasonic cleaning machine for 5 min, dried with cold air, and placed in a drying oven. The detailed process is shown in Figure 4.

#### 2.3.2. Electrolytic Polishing and Metallographic Microstructure Observation

The microscopic metallographic structures of the deformation layer of the original samples and the USRT samples were observed using a metallographic microscope (IE500M, Beijing Shun Yu new optical instrument Co., LTD, Beijing, China). The cross-sections of the samples were chemically corroded. The corrosion solution, i.e., a mixed acid solution (mass percentage: nitric acid 5% + hydrofluoric acid 17% + deionized water 78%), was used for approximately 15 s; then, the samples were washed with water to remove the corrosive liquid and finally cleaned with acetone in an ultrasonic cleaning machine and dried. The preparation process of the electropolished samples was the same as that for metallographic samples, except that the electropolished samples were connected to wires. The specific electrolytic polishing solution used for the samples was 10% perchloric acid and 90% alcohol. The electrolyte temperature was kept at −28 °C, and the voltage was 25 V.

#### 2.3.3. Surface Morphology

Laser confocal scanning microscopy (LEXT OLS4000, Olympus Corporation, TKY, JAPAN) was used to observe the surface roughness and morphology of the original sample and the outer circular surface of the USRT sample. The semiconductor laser with a wavelength of λ = 405 nm was selected with a two-dimensional (2D) resolution of 0.12 mm and a Z-axis resolution of 0.01 mm.

#### 2.3.4. SEM Analysis

The cross-sectional microstructure morphology of the original sample and the USRT sample was observed with field emission scanning electron microscopy (FESEM, Quanta FEG 650, FEI Company, Hillsboro, OR, USA) with an acceleration voltage of 15–20 kv. To characterize the morphology of the gradient deformation layer of the USRT samples, the morphology was observed by using secondary electron imaging, which is sensitive to the orientation of the grain morphology. For the analysis of the secondary precipitates, the inclusion of elements, and the content of aluminum alloys, energy-dispersive X-ray spectroscopy (EDS) with field emission scanning electron microscopy (FESEM, Quanta FEG 650, FEI Company, Hillsboro, OR, USA)was used.

#### 2.3.5. TEM Analysis

**Figure 5:**
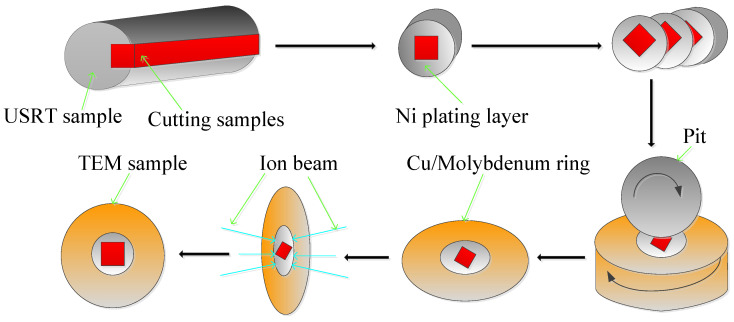
Schematic diagram of TEM sample preparation process.

The high resolution transmission electron microscope (TEM, FEI Tecnai, G2F20, FEI Company, Hillsboro, OR, USA) was used to observe the microstructure of the cross-section of the gradient deformation layer. The TEM samples were processed using the FEI Helios Nanolab 600i dual-beam micro-nano processing imaging system with a focused-ion beam (FIB) function. The TEM sample preparation process is as follows: (1) Sampling was carried out by cutting the rectangular pieces to a dimension of 2.5 mm × 2.5 mm × 5 mm from the original sample with EDM. (2) The sample was electroplated with a 5 μm thick pure platinum protective layer. Before electroplating, the cutting surface of the sample was polished and ultrasonically cleaned. The activation was conducted with acetone. (3) The electroplated sample was cut into slices with a thickness of 0.5 mm. (4) Thin section samples were polished step-by-step up to a thickness of 30 μm in varied sizes using water and sand with coarse to fine grains. (5) A standard TEM sample with a diameter of 1 mm was prepared using a pitting machine, and then, thin-sheet specimens were glued to the molybdenum ring with epoxy resin adhesive, and finally, standard TEM specimens with a diameter of 1 mm were achieved. (6) The FEI Helios Nanolab 600i dual-beam micro–nano machining system(FIB, FEI Helios Nanolab 600i, FEI Company, Hillsboro, OR, USA) was used to thin out the sample with a FIB. The detailed process is shown in Figure 5.

#### 2.3.6. XRD Characterization

In this study, the phase constitutions were characterized with the X-ray diffractometer(XRD, Bruker/D2 PHASER, Bruker AXS, Karlsruhe, Germany). The XRDwith Cu–Kα radiation was used by setting the X-ray wavelength Kαλ = 0.154056 nm at the power of 300 W, test voltage of 35 kV, current of 30 mA, and scanning range (θ) 20–90°. The diffraction data were collected by using the Jade 6.5 analysis software to figure out the peak position, decompose and fit the peak shape, and calculate the unit cell parameters, grain size, and lattice distortion rate.

## 3. Results and Discussion

### 3.1. The Original Structure of AA7075

**Figure 6:**
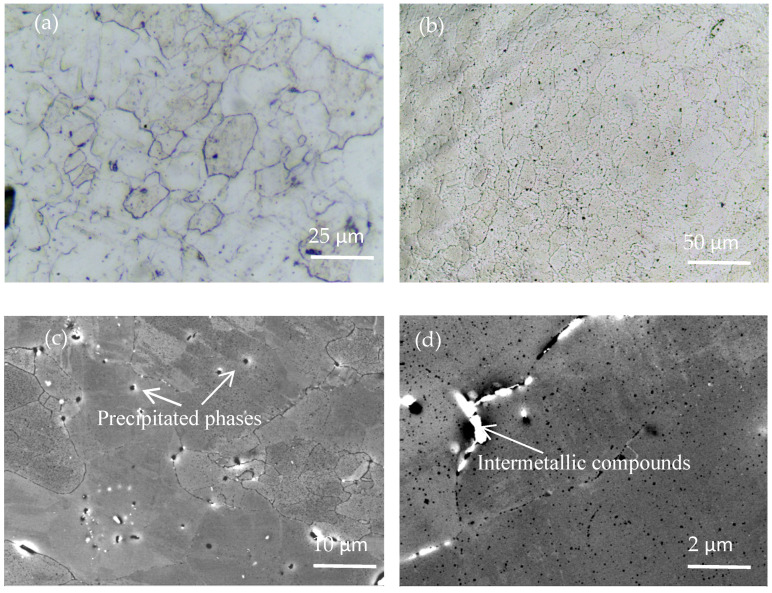
The original microstructure of AA7075 aluminum alloy: (**a**) Chemical etching metallographic optical morphology, (**b**) Metallographic morphology of electrolytic polishing, (**c**) SEM morphology of microstructure, and (**d**) Local high magnification morphology of grain boundaries of microstructures.

The microstructure morphology of the original material (AA7075) after forging and T6 heat treatment (Solution treatment + artificial aging) is shown in Figure 6. Figure 6a,b show that the original material consists of coarse grains with irregular shapes and uneven grain size. Figure 6c,d illustrate that the microstructure of the original material is composed of an α-aluminum matrix, and the precipitation strengthening phase is dispersed in the grain and grain boundary. According to the literature [28], second-phase particles are mainly composed of magnesium-rich phase (η, $\eta^{\prime }$) and iron-based compounds (Al_7_Cu_2_Fe, Al_3_Fe). The η phase is the MgZn_2_ equilibrium phase, which is not coherent with the α-aluminum matrix and usually exhibits a flaky structure. The $\eta^{\prime }$ phase is the transition phase of MgZn_2_ and usually produces a disc-shaped structure. Iron-based compound particles are silvery-white and do not show a fixed shape. After the AA7075 is treated with T6 heat treatment, larger recrystallized grains appear, and the content of the undissolved phase is reduced. The uncrystallized region is composed of subgrains with small angle grain boundaries and fine recrystallized grains with a large angle.

### 3.2. Surface Morphology of the Sample

**Figure 7:**
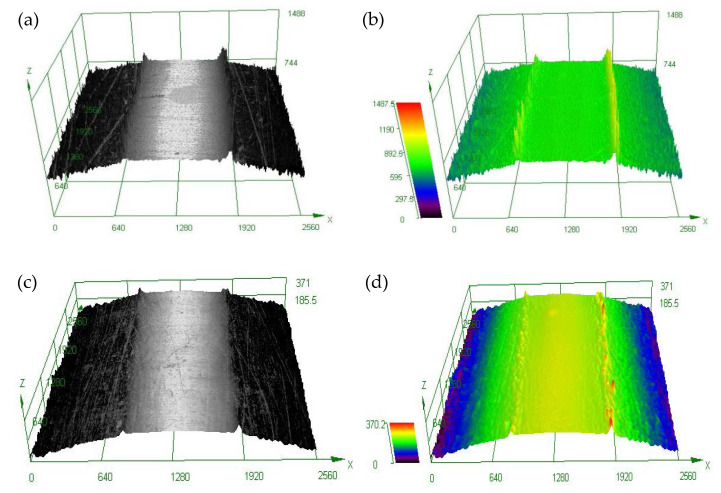
Surface morphology of original sample and USRT sample (Note: The unit of coordinate value in the figure is nm): (**a**) 3D morphology of the original sample, (**b**) 3D contour of the original sample, (**c**) 3D morphology of USRT sample, and (**d**) 3D contour of USRT sample.

Figure 7a shows the 3D topography of the surface. Convex and concave grooves are observed on the surface of the original sample, and numerous machining traces are left by the machining tools along the vertical direction of the sample axis. Compared with the original sample, the surface of the USRT sample is brighter; the scratches, grooves, and other defects left by the mechanical machining and polishing are significantly reduced (Figure 7c), and the surface finish of the USRT samples is significantly improved. Comparative analysis of the 3D contour lines in Figure 7b,d indicates a significant reduction in the surface roughness of the USRT samples. The USRT process applies a certain amplitude of high-frequency mechanical vibration along the normal direction of the workpiece surface through the machining head. Under certain feeding conditions, the working head transmits the rolling pressure and ultra-high frequency impact vibration to the surface of the rotating machinery spare parts. Under the dual load of ultra-high frequency impact energy and static load rolling, the microplastic deformation of the surface layers is produced. These results indicate the “ironing peak and filling valley” similar to the mirror treatment effect. Continuous rolling can generate residual compressive stress on the surface of AA7075, which can press the surface processed in the earlier pass and weld some small defects on the surface so that the uniformity of the processed surface tends to be consistent. The testing results of the surface roughness tester (Elcometer 7062) show that the average surface roughness value of the experimental samples decreased ($R_{a}$) from 2.25 to 0.45 μm. This value is lower than that of other surfaces, such as the UIT, SMAT [29], SMGT [30], SMRT [31], and other surface-treatment methods.

### 3.3. XRD Analysis

**Figure 8:**
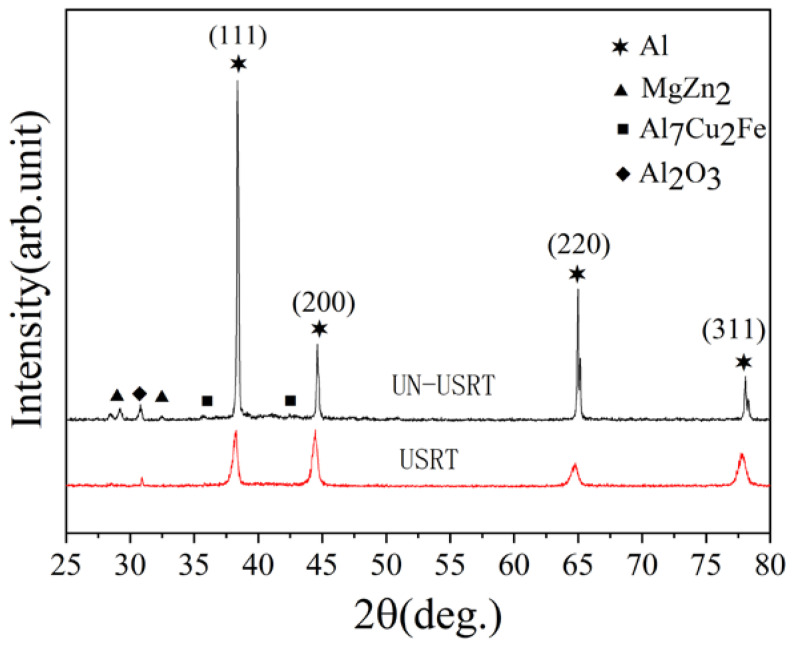
XRD patterns of the surface of the original and USRT samples.

Figure 8 shows the XRD patterns of the surface of the original and the USRT samples. The results indicate that the original sample shows the diffraction of the second phase precipitates (MgZn_2,_ Al_7_Cu_2_Fe) in the XRD pattern. However, due to the small proportion of the second-phase precipitate particles in the aluminum matrix, its diffraction peak is weak, and the diffraction peak of the second-phase precipitate particles cannot be observed on the surface of the USRT sample. For both samples, the main characteristic phase of the diffraction peaks corresponds to the aluminum matrix phase, which clearly indicates that the USRT does not lead to the phase transition in the surface deformation layer region. Compared with the original sample, the full width at half maximum (FWHM) of the Bragg diffraction peak of the USRT sample is significantly widened, which can be assigned to the increase in surface grain refinement and lattice distortion caused by the USRT. These results are consistent with the results obtained by Pandey et al. [25,26].

Lattice distortion and grain refinement have an enormous influence on the mechanical properties of the material; thus, the changes in grain size and lattice distortion are significant for the surface nanocrystallization to improve the surface hardness and wear resistance of aluminum alloys. In general, the smaller the grain size, the greater the lattice distortion and the greater the hardness of the material; moreover, the increase in hardness can usually improve the wear resistance. Therefore, the grain size was calculated by using the Bragg diffraction peak FWHM of the face-centered aluminum alloy phase, and the effect of grain refinement degree and lattice distortion on the FWHM of the diffraction peak was explored. The quantitative calculation was conducted according to the Scherer–Wilson equation as follows:

(1) $$D_{c}=\frac{K\lambda }{B\cos\theta }$$

where $D_{c}$ is the effective grain size of the crystal surface normal line (nm), *K* is the shape coefficient (for cubic grain *K* is 0.89), λ is the incident ray wavelength, θ is the incident angle, and *B* is the FWHM of the diffraction peak. The internal lattice distortion of the USRT sample is calculated according to the Williamson–Hall formula:

(2) $$B\cos\theta =[\frac{K\lambda }{D_{c}}]+[4\varepsilon \sin\theta ]$$

where ε is the root mean square of microstrain (lattice distortion). The average grain size and lattice distortion obtained with calculation are presented in Table 4.

### 3.4. Surface Microstructural Features of the Gradient Structure

The overall effect of the USRT treatment on the surface microstructure of 7075 aluminum alloy can be described as follows: applying a load on the surface of the sample generates a stress field, and the stress reaches a certain level, causing plastic deformation on the surface in different directions. Plastic deformation causes the grains and second-phase precipitated particles of the aluminum alloy to continuously break, and the degree of second-phase particle fragmentation is related to the degree of deformation; as the plastic deformation becomes more severe, the greater is the degree of fragmentation of the second-phase particles in the deformation zone. The deformation first occurs on the surface layer of the 7075 aluminum alloy. With the extension of time and continuous USRT treatment, the plastic deformation becomes greater as it approaches the surface layer, and the second-phase particles are more easily fragmented until they are refined to the nanometer level. The deformation degree of the surface layer is different from that far from the surface layer, and the refinement degree of the microstructure is also different; therefore, the microstructure near the surface layer of the 7075 aluminum alloy presents a gradient distribution along the thickness direction.

**Figure 9:**
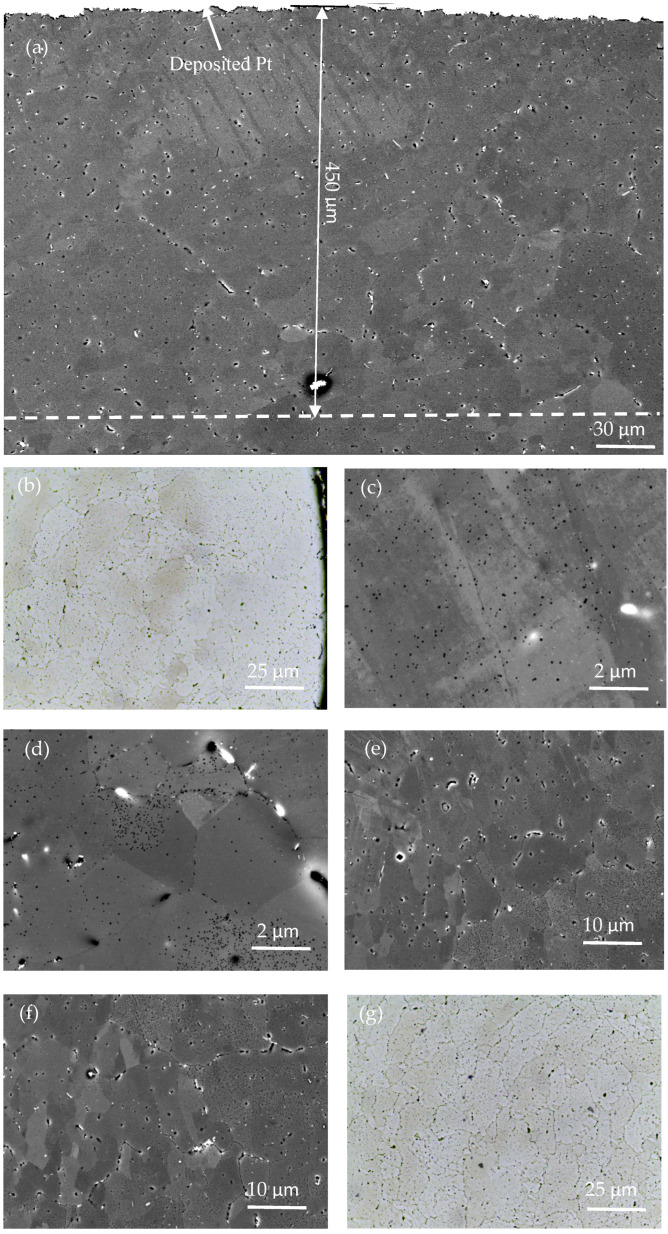
The microstructure morphology of AA7075 aluminum alloy with gradient structure at different depths along the cross-section: (**a**) SEM image showing morphology of the microstructure within 450 μm from the treated surface, (**b**) Electrochemical polishing metallographic morphology of surface region, (**c**) Local SEM high-magnification image showing morphology of surface region, (**d**) High-magnification SEM image showing morphology of the local area of the undeformed layer, (**e**) SEM image of microstructure within 250 μm from the treated surface, (**f**) SEM image of microstructure of the undeformed layer, and (**g**) Electropolished metallographic morphology of the undeformed layer region.

Figure 9 shows the microstructure of the cross-section of the USRT sample. Figure 9a,b demonstrate that a plastic deformation and grain refinement layer with a thickness of approximately 450 μm is formed on the surface of the sample after USRT. Pandey et al. [25,26] reported that the thickness of the deformation layer was significantly larger than that of the AA7075 treated with USSP, but it was smaller than that of the gradient deformation layer (reported by Qi Wang [32] and Huang Haiwei [31]) by applying the SMGT method on the surface of pure titanium and pure copper, respectively. This result is mainly attributed to the fact that pure copper and pure titanium show better plasticity and better plastic deformation ability, and the surface layer of the sample after the SMGT can obtain a better grain refinement effect and thicker deformation layer depth. However, for AA7075, the plastic deformation ability is poor, and it is difficult to refine the surface grains and form a thicker surface deformation layer. Zhang and coworkers [16,33] obtained similar conclusions when using the SMRT to process the ultra-high-strength AISI 52100.

In the USRT process, the working head was used to apply a certain amplitude of high-frequency mechanical vibration to the surface of the sample along the normal direction of the surface, and the sample was subjected to high stress and strain gradients along the direction from the treated surface to the core. From the treated surface of the sample to 20 μm, the grain structure produced severe plastic deformation through dislocation slip, and the degree of fragmentation of the grain was significant. The visible grain boundaries were destroyed or even disappeared, and the grain boundary and interfacial structure became blurred. TEM could only characterize the microstructure deformation characteristics of this region. The observation of the treated surface area (Figure 9c) and the local enlarged image of the undeformed coarse-grained area (Figure 9d) indicates that the grain boundaries of the treated surface and part of the coarse precipitation-strengthening precipitates in the grains are significantly reduced, and the microstructure uniformity is significantly improved. At the subsurface deformed layer 250 μm away from the treated surface (Figure 9e), the grain shape and size exhibited a clear transition gradient, and the grain boundary changed from fuzzy to clear with the increase in the depth from the surface. What Figure 9f,g exhibit is that, when the depth from the treatment surface is greater than 450 μm, the microstructure morphology is an undeformed coarse-grained matrix.

**Figure 10:**
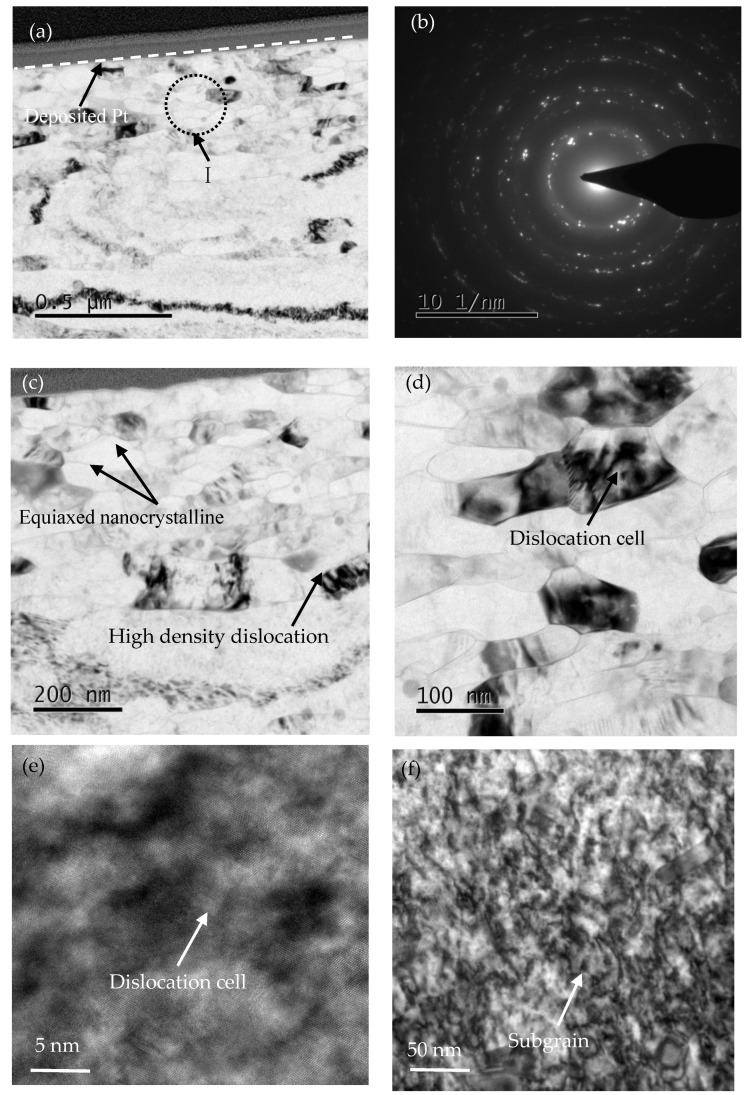

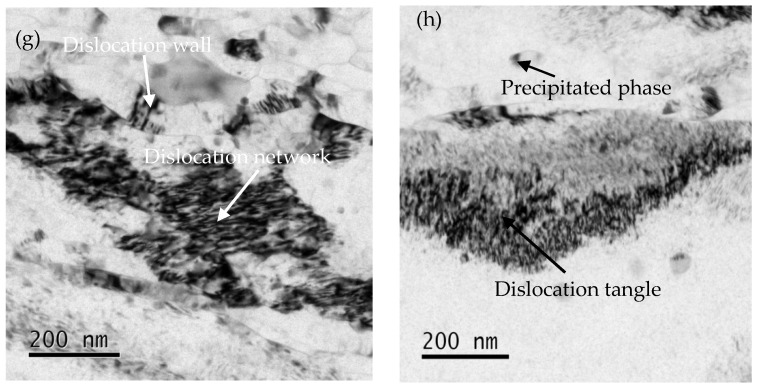
TEM image showing morphology of cross-section of USRT sample: (**a**) Morphology of surface area, (**b**) SAED map of position I in (**a**), (**c**) TEM image of surface to inner 15–20 μm region, (**d**) The magnified morphology of the local area of the surface deformation layer, (**e**) The magnified morphology of the dislocation configuration at approximately 60 μm from the treated surface, (**f**) The local magnified morphology of the subgrain region at approximately 60 µm from the treated surface, (**g**) The localized dislocation configuration at approximately 120 μm from the treated surface (enlarged morphology), and (**h**) Locally enlarged morphology of dislocation configuration at approximately 120 μm from the treated surface.

Figure 10 shows the TEM images, exhibiting the microstructure and diffraction pattern of the USRT sample at different depths from the surface. Figure 10a,c,d exhibit that the grain size of the USRT sample is distributed in a gradient pattern. The dimensions vary from the nanoscale to the microscale. With the increase in the depth from the treated surface, the grain size from the surface to the inside changes from nanometer scale to micron scale. At the same time, the dislocations caused by the severe plastic deformation on the surface can be observed, and the dislocation density gradually decreases from the surface to the inside. In the range of 15–20 μm from the treated surface to the interior core (Figure 10c), the microstructure is composed of nanocrystals. Most nanocrystals show sharp grain boundaries, and some grains are filled with high-density dislocations. The statistical results of the TEM measurements show that the average grain size of the nanocrystals is approximately 70 nm, which is similar to the average grain size of the surface grain (approximately 77 nm) estimated with the Scherer–Wilson method via XRD analysis (discussed in Section 3.3). At the same time, the selected electron diffraction pattern (SAED) of the corresponding region of the nanocrystalline layer showed a circular distribution (Figure 10b), which specified that the randomly oriented equiaxed nanocrystals were formed on the surface of the sample after the USRT. The selected area of the electron diffraction pattern showed a discontinuous and discrete distribution, illustrating that the surface layer of the sample after the USRT contained subgrains. Moreover, the second-phase precipitation particles were not observed in the local area of the surface deformation layer enlarged in Figure 10d, which was due to the severe plastic deformation with a high strain rate introduced by the USRT in the surface region of the sample, resulting in the fragmentation of precipitated particles of the second phase in the α-aluminum matrix. In the region of the subsurface deformation layer approximately 60 μm away from the treated surface (Figure 10e,f), the high-density dislocation instigated some grains to form dislocation cells and subgrains, and some grain boundaries were blurred, which indicated that high internal stress and lattice distortion occurred at grain boundaries during severe plastic deformation. The dislocation distribution was uniform, which indicates that the aluminum matrix was uniformly cut and refined by the dislocation movement. At the same time, the contrast of crystal lining degree was not uniform, which showed high internal stress and elastic deformation in the lattice. The deformed layer region approximately 120 μm away from the treated surface, as shown in Figure 10g,h, exhibits the formation of high-density dislocations, dislocation walls, and a network of dislocations by the entanglement of dislocations in some ingrain and grain boundaries. This result can be attributed to the fact that the increase in internal stress imposed by the USRT led to the proliferation of dislocations. Furthermore, numerous high-density dislocations underwent inhomogeneous aggregation, forming dislocation walls, dislocation networks (Figure 10g), and dislocation entanglement (Figure 10h), resulting in a reduced degree of uniform cutting of the aluminum matrix. Therefore, the grain size in this region was larger than that in the region with a small distance from the surface. The above-mentioned analysis confirmed that the main reasons for the gradient change in grain size after the USRT were the internal stress caused by durable plastic deformation and the cutting effect of dislocation on the aluminum matrix.

### 3.5. Microstructural Evolution Analysis of Surface Layer with Gradient Structure

**Figure 11:**
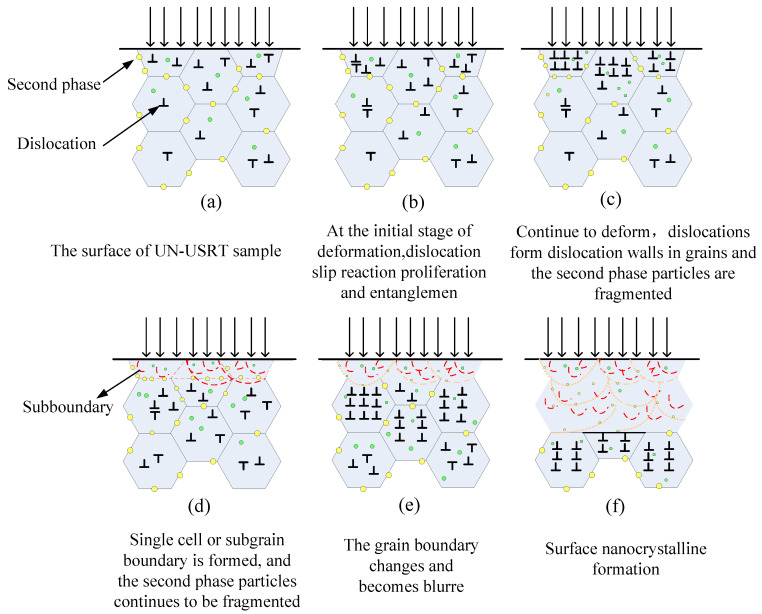
Schematic showing the evolution mechanism of microstructure refinement of AA7075 subjected to USRT.

The surface grain refinement process of the AA7075 sample treated with the USRT is shown in Figure 11. The crystal structure of aluminum alloy is a face-centered cubic structure, the stacking fault energy is high, and the deformation acts through dislocations [34]. Dislocation slip dominates the grain refinement process, and dislocation segmentation and second-phase precipitated particle fragmentation are the main mechanisms of its grain refinement.

A similar refinement mechanism was also observed for the surface grains of AA7075 obtained with the USSP treatment [25,26]. When the USRT is not applied, as described in Section 3.1, second phases exist in the microstructure of AA7075 near the surface layer, which may be distributed in the grain and at the grain boundary, and there are also dislocations in the grain. Under the condition of the USRT, part of the energy applied to the aluminum alloy with the USRT is used for plastic deformation, and part of the energy is stored, which exerts an effect on the dislocation.

When the rolling time is short, as shown in Figure 11b, the plastic deformation, dislocation slip, annihilation, or new dislocations and tangles occur when dislocations gather excessively and cause the distortion of the crystal lattice. When the deformation reaches a certain level, as shown in Figure 11c, the slip of dislocations to the grain boundaries is hindered, resulting in stress concentration. When the stress concentration and the applied force make the second phase crumble, the second phase in the grain is broken into small particles. The blockage of dislocations at the grain boundary prevents the dislocation source from releasing dislocations; however, it cannot prevent the aggregation of dislocations inside the grains. When the number of dislocations is large, rearrangement and entanglement occur inside the grains, forming dislocation walls. Dislocation walls divide a grain into dislocation cells.

The size of the dislocation cell (*L*) is related to the shear force (*τ*) produced by the grain, the shear modulus (*G*) of the aluminum alloy, and the Burger vector (b) of the dislocation. The relationship between the factors is expressed by *L* = 10*Gb*/*τ* [35]. Owing to the different distances from the surface of AA7075, the deformation degree and shear stress are different, and the size of the dislocation cell is also different. The closer the dislocation to the surface of AA7075, the larger the shear stress, and the size of the dislocation cell can reach up to the nanometer level. During this process, the number of dislocations affects the size of the dislocation cell. The higher the number of dislocations, the more the dislocations get tangled together, and the more the dislocation cells with smaller size are generated. Too many dislocations and entanglements lead to an increase in the energy of the system. To reduce the energy, the dislocations near the dislocation wall and at the dislocation entanglement get rearranged, react, and annihilate again. The dislocation wall and entangled dislocations are transformed into unit cells or subgrain boundaries, as shown in Figure 11d. The appearance of unit cells and subgrain boundaries reduces the dislocation density and the system energy. If the strain continues to increase, dislocations are generated continuously, which can be hindered at the subgrain boundary and at the grain boundary, or the grain boundary is rotated by aggregation or annihilated by the reaction. This further leads to the increase in the atomic arrangement and orientation difference between the two adjacent grain boundaries. In the case of large deformations, the energy accumulated inside the material further increases, the original grain boundary of AA7075 also begins to rotate, the phase difference between two adjacent grains becomes maximum, and finally, the grain boundary attains the least size. This breaks the second phase, and the original grain boundary is partially disintegrated (Figure 11e). When the strain of the grain layer near the surface reaches a certain degree, dislocation walls are formed. As a result, bright white fragments can be observed at the grain boundaries treated with the USRT (Figure 11d). In the case of high strain and high strain rate of the USRT, the dislocation density generated on the surface is extremely high, and the dislocation walls and dislocation cells are small. After the dislocation walls and dislocation, cells develop into grain boundaries and reach nanoscale size; thus, a nanocrystalline structure is developed (Figure 11f), and the corresponding second- and third-layer grains near the surface are transformed into subgrain boundaries and dislocation walls. With the further increase in deformation, they are transformed into nanocrystals or microcrystals. Therefore, the experimental samples form a nanocrystalline/ultrafine grain layer, a subsurface deformed grain layer, and a core coarse-grained matrix layer in sequence, which indicates that the grain size is distributed according to a gradient law.

## 4. Conclusions

By optimizing the processing parameters, a gradient structure deformation layer was prepared on the surface of a 7075 aluminum alloy sample with USRT. The microstructure evolution mechanism at different depths was characterized with LSCM, SEM, XRD, and TEM. The important research findings include:

(1) After USRT surface treatment, the average roughness of the sample decreased ($R_{a}$) from 2.25 μm (before treatment) to 0.45 μm (after treatment), which was lower than that of other surface treatment methods, such as USSP, UIT, and SMAT. After the treatment, the surface microstructure of the samples did not undergo a phase change, and the second-phase particles dispersed in the surface layer underwent fragmentation in the α-aluminum matrix.

(2) The surface of the USRT sample formed a 450 μm thick deformation layer with a gradient distribution of grain size. The surface layer was composed of nanocrystalline/ultrafine crystals, and the inner part was composed of an undeformed coarse crystal matrix. The average grain size of the surface layer was 70 nm, and the grain was randomly oriented. The thickness of the nanocrystalline layer was approximately 15–20 μm. For aluminum alloy, for a higher-level fault energy material, the surface deformation layer formed with the USRP treatment was thicker than that with traditional SP and SMAT techniques. The results of this study lay a foundation for future studies to investigate mechanical properties, fatigue properties, surface friction, and wear properties.

(3) The surface deformation mechanism of AA7075 aluminum alloy with high-level fault energy is high strain rate induced dislocation slip. A large number of accumulated dislocations interact with dislocations and second-phase precipitated particles, resulting in dislocation annihilation and recombination to form new dislocation configurations (dislocation cells, subgrains). The continuous cutting of dislocations refines dislocation cells and subgrains, and the interaction of multiple dislocation slip systems cuts and breaks down the second-phase particles, which is the main mechanism for grain refinement near the surface layer. This research provides a theoretical foundation for studying the surface grain refinement and nanocrystallization of the same type of precipitation-strengthened aluminum alloy.

## Figures and Tables

**Table 1:** Chemical composition of test material (wt.%).

Al	Fe	Si	Mn	Cu	Mg	Cr	Zn	Ti
Bal.	0.29	0.12	0.05	1.60	2.50	0.18	5.50	0.02

**Table 2:** Mechanical properties of test material.

0.2% Proof Strength	Tensile Strength	Elongation	Elasticity Modulus	Density	Hardness
465 MPa	550 MPa	12%	71 GPa	2.81 g⋅cm^−3^	128HV

**Table 3:** The process parameters for USRT of AA7075.

Impact Head	Spindle Speed	Static Pressure	Feed Speed	Output Frequency	Amplitude of Working Head	Pass
WC/Co (spherical)	110 r⋅min^−1^	400 N	0.1 mm⋅r^−1^	28.62 KHz	7 μm	3

**Table 4:** Average grain size and lattice distortion of the original sample and USRT sample.

Specimen Type	Average Grain Size (nm)	Average Lattice Distortion (%)
UN-USRT	155	0.069
USRT	77	0.322

## Data Availability

Not applicable.
